# The Menkes and Wilson disease genes counteract in copper toxicosis in Labrador retrievers: a new canine model for copper-metabolism disorders

**DOI:** 10.1242/dmm.020263

**Published:** 2016-01-01

**Authors:** Hille Fieten, Yadvinder Gill, Alan J. Martin, Mafalda Concilli, Karen Dirksen, Frank G. van Steenbeek, Bart Spee, Ted S. G. A. M. van den Ingh, Ellen C. C. P. Martens, Paola Festa, Giancarlo Chesi, Bart van de Sluis, Roderick H. J. H. Houwen, Adrian L. Watson, Yurii S. Aulchenko, Victoria L. Hodgkinson, Sha Zhu, Michael J. Petris, Roman S. Polishchuk, Peter A. J. Leegwater, Jan Rothuizen

**Affiliations:** 1 Department of Clinical Sciences of Companion Animals, Faculty of Veterinary Medicine, Utrecht University, Yalelaan 104, 3584 CM Utrecht, The Netherlands; 2 The WALTHAM Centre for Pet Nutrition, Waltham-on-the-Wolds, Melton Mowbray, Leicestershire, LE14 4RT, UK; 3 Telethon Institute of Genetics and Medicine (TIGEM), Via Campi Flegrei 34, 80078 Pozzuoli (NA), Italy; 4 TCCI Consultancy BV, Cicerolaan 1, 3584 AJ Utrecht, The Netherlands; 5 Department of Pediatrics, Molecular Genetics Section, University of Groningen, University Medical Center, Antonius Deusinglaan 1, 9713 AV Groningen, The Netherlands; 6 Department of Pediatric Gastroenterology, Wilhelmina Children's Hospital, University Medical Center, Lundlaan 6, 3584 EA Utrecht, The Netherlands; 7 Novosibirsk State University, 630090 Novosibirsk, Russia; 8 Institute of Cytology and Genetics, 630090 Novosibirsk, Russia; 9 Department ofBiochemistry, University of Missouri, Columbia, MO 65211, USA; 10 The Christopher S. Bond Life Science Center, University of Missouri, Columbia, MO 65211, USA; 11 Nutrition and Exercise Physiology, University of Missouri, Columbia, MO 65211, USA

**Keywords:** ATP7A, ATP7B, Dog, Liver

## Abstract

The deleterious effects of a disrupted copper metabolism are illustrated by hereditary diseases caused by mutations in the genes coding for the copper transporters ATP7A and ATP7B. Menkes disease, involving *ATP7A*, is a fatal neurodegenerative disorder of copper deficiency. Mutations in *ATP7B* lead to Wilson disease, which is characterized by a predominantly hepatic copper accumulation. The low incidence and the phenotypic variability of human copper toxicosis hamper identification of causal genes or modifier genes involved in the disease pathogenesis. The Labrador retriever was recently characterized as a new canine model for copper toxicosis. Purebred dogs have reduced genetic variability, which facilitates identification of genes involved in complex heritable traits that might influence phenotype in both humans and dogs. We performed a genome-wide association study in 235 Labrador retrievers and identified two chromosome regions containing *ATP7A* and *ATP7B* that were associated with variation in hepatic copper levels. DNA sequence analysis identified missense mutations in each gene. The amino acid substitution ATP7B:p.Arg1453Gln was associated with copper accumulation, whereas the amino acid substitution ATP7A:p.Thr327Ile partly protected against copper accumulation. Confocal microscopy indicated that aberrant copper metabolism upon expression of the ATP7B variant occurred because of mis-localization of the protein in the endoplasmic reticulum. Dermal fibroblasts derived from ATP7A:p.Thr327Ile dogs showed copper accumulation and delayed excretion. We identified the Labrador retriever as the first natural, non-rodent model for *ATP7B*-associated copper toxicosis. Attenuation of copper accumulation by the *ATP7A* mutation sheds an interesting light on the interplay of copper transporters in body copper homeostasis and warrants a thorough investigation of *ATP7A* as a modifier gene in copper-metabolism disorders. The identification of two new functional variants in ATP7A and ATP7B contributes to the biological understanding of protein function, with relevance for future development of therapy.

## Abstract (precis)

**Summary:** Labrador retrievers with hereditary copper toxicosis are a useful new model for copper-metabolism disorders.

## INTRODUCTION

Copper is an essential trace element for a wide range of biochemical processes in the body. The P-type copper-transporting ATPases ATP7A (Vulpe et al., 1993) and ATP7B (Bull et al., 1993) are crucial for copper homeostasis. At the cellular level, these proteins have a biosynthetic role in the *trans*-Golgi network, where they facilitate incorporation of copper into proteins. Furthermore, they prevent toxic accumulation of cellular copper by redistribution to a vesicular compartment, resulting in excretion of copper through either the apical membrane (ATP7B) or the basolateral membrane (ATP7A). This redistribution involves metal-binding domains (MBDs) in cytoplasmic domains of the proteins and is regulated in part by their phosphorylation state (Voskoboinik et al., 2002).

Body copper homeostasis is maintained by balancing the rates of dietary copper absorption and biliary copper excretion. ATP7A is highly expressed in enterocytes, where it is involved in copper uptake across the basolateral membrane into the portal circulation for delivery to the liver, where copper is stored (Linder and Hazegh-Azam, 1996). Besides its function in intestinal epithelial cells, ATP7A was recently identified to play an important role in hepatic copper mobilization in response to copper demands of peripheral tissues (Kim et al., 2010). Excess hepatic copper is excreted into the bile and requires expression of functional ATP7B in hepatocytes (Gitlin, 2003).

The severe effects of copper-metabolism imbalance are illustrated by inherited disorders resulting from mutations in *ATP7A* and *ATP7B*.

Mutations in *ATP7A* result in a fatal, X-linked copper-deficiency disorder in infants known as Menkes disease. The disease is characterized by cerebral and cerebellar degeneration, failure to thrive, coarse hair and connective tissue abnormalities (Kaler, 2011).

Wilson disease results from mutations in *ATP7B* (Gitlin, 2003) and is associated with copper accumulation in the liver and secondarily in the brain, resulting in hepatic cirrhosis and neuronal degeneration. The age of onset and the clinical manifestations vary greatly between individuals affected by Wilson disease. This lack of genotype-phenotype correlation might be influenced by currently unidentified genetic modifiers. Other hereditary diseases leading to hepatic copper accumulation in infants include Indian childhood cirrhosis (Tanner, 1998) and endemic Tyrolean infantile cirrhosis (Müller et al., 1996). In these diseases, the causal genes are currently unknown, and dietary copper intake is believed to contribute significantly to disease progression.

In order to develop new treatment strategies for copper-metabolism disorders, several rodent models were investigated, including natural models, such as the mottled mouse (Grimes et al., 1997), the toxic milk mouse (Theophilos et al., 1996) and the Long–Evans cinnamon rat (Li et al., 1991), in addition to *ATP7A* (Wang et al., 2012) and *ATP7B* knockouts (Buiakova et al., 1999). Although rodent models are invaluable for studying diseases, the dog as a large-animal model represents a unique translational bridge between rodents and humans. The best-characterized canine model for copper toxicosis is the Bedlington terrier, in which severe hepatic copper accumulation is caused by a deletion in the *COMMD1* gene (Van de Sluis et al., 2002). However, convincing evidence of involvement of the *COMMD1* gene in human copper-metabolism disorders is lacking (Coronado et al., 2005; Lovicu et al., 2006; Müller et al., 2003).

The Labrador retriever dog breed was recently characterized as a new mammalian model for copper toxicosis, distinct from *COMMD1-*associated autosomal recessive copper toxicosis. In predisposed Labrador retrievers, hepatic copper accumulation induces hepatic cirrhosis, usually in middle-aged dogs. Female Labrador retrievers are at increased risk for the disease (Fieten et al., 2012b).

The genes underlying the disease in Labrador retrievers were not known. We hypothesized that identification of these genes would contribute to the understanding of copper-metabolism biology in dogs and other mammals. Given that the copper genome is highly conserved across species, this study might reveal genes that are candidate modifiers in human copper-metabolism disorders. Furthermore, elucidating the genetic background of copper toxicosis in Labrador retrievers contributes to the characterization of this new model for copper-metabolism disorders, necessary to exploit its possibilities fully.

Whereas gene-mapping studies in human complex diseases require thousands of affected individuals and millions of single nucleotide polymorphism (SNP) markers, smaller numbers suffice for a genome-wide association study (GWAS) of inbred pedigree dogs owing to the reduced genetic and phenotypic heterogeneity. Therefore, the dog is a useful model for identifying genes in naturally occurring complex hereditary diseases that are phenotypically similar in dogs and humans (Boyko, 2011).

The aims of the present study were to identify gene variants involved in copper toxicosis in the Labrador retriever and to provide biological evidence for their involvement in the disease.

## RESULTS

### Dogs

The initial GWAS cohort consisted of 235 Labrador retrievers. The independent replication cohort consisted of 59 Labrador retrievers. The median copper score in the 294 Labrador retrievers included in the study was 1.5 (range 0-5). A summary of characteristics of the dogs, including age, sex and hepatic copper scores, is provided in Table S1.

### Electron microscopic evaluation of copper toxicosis in Labrador retrievers

**Fig. 1.**
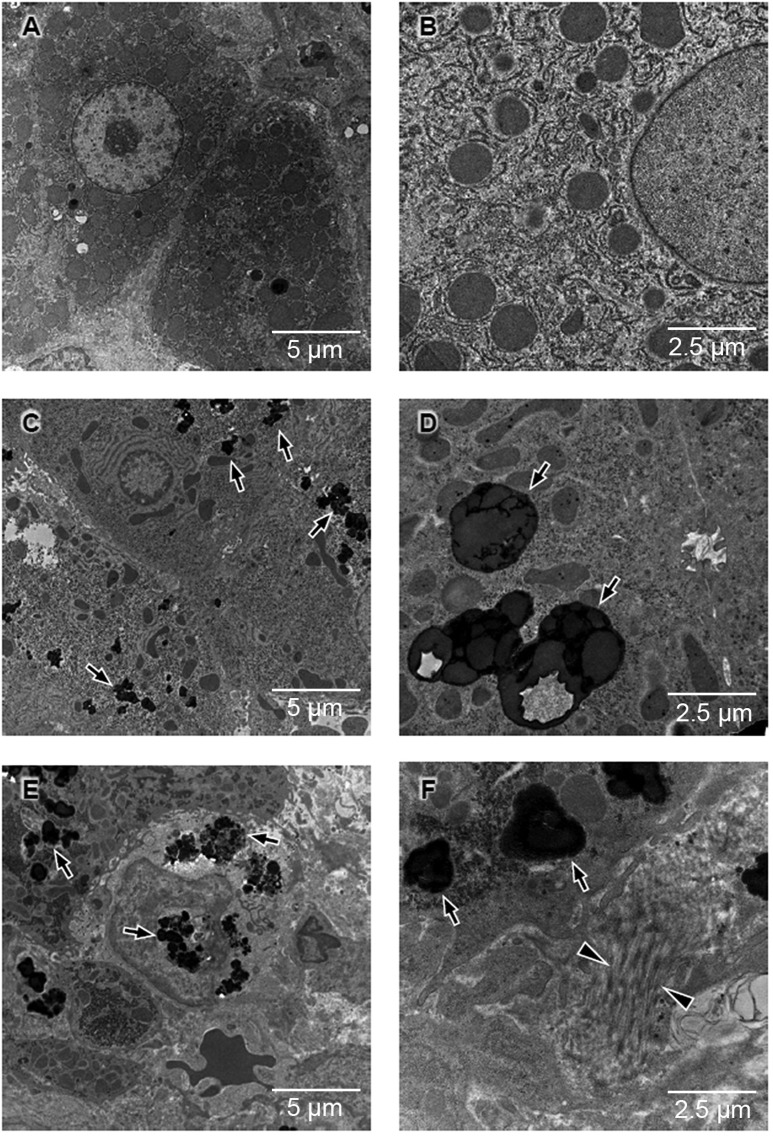
**Hepatic ultrastructure in Labrador retrievers with elevated copper levels.** (A,B) Low (A) and high (B) magnification of thin sections from the liver reveal regular organization of the hepatic tissue in a 1-year-old male cross-breed (control dog, hepatic copper 250 mg/kg dwl). (C,D) Ultrastructure of an 11-year-old female Labrador retriever [affected dog, hepatic copper 756 mg/kg dry weight liver (dwl)] exhibits wide areas with accumulation of electron-dense material (C, arrows), which at higher magnification appear as copper-laden lysosomes comparable to what is observed in humans with Wilson disease (D, arrows). (E,F) Ultrastructure of a liver biopsy of a 12-year-old female Labrador retriever (affected dog, hepatic copper 1445 mg/kg dwl); some areas of the liver show severe disorganization of the tissue architecture (E), with massive electron-dense inclusions (E,F; arrows). Arrowheads in F indicate bundles of collagen fibres as a sign of extensive fibrosis.

To complement the phenotype description of copper toxicosis in Labrador retrievers, liver biopsies of two Labrador retrievers affected with copper toxicosis and a control were assessed by electron microscopy. Ultrastructural changes in the biopsies of the affected dogs corresponded to copper-laden lysosomes, comparable to what is recognized by electron microscopy of the liver of human individuals with Wilson's disease (Fig. 1).

### Genome-wide association study of copper toxicosis in Labrador retrievers

**Fig. 2.**
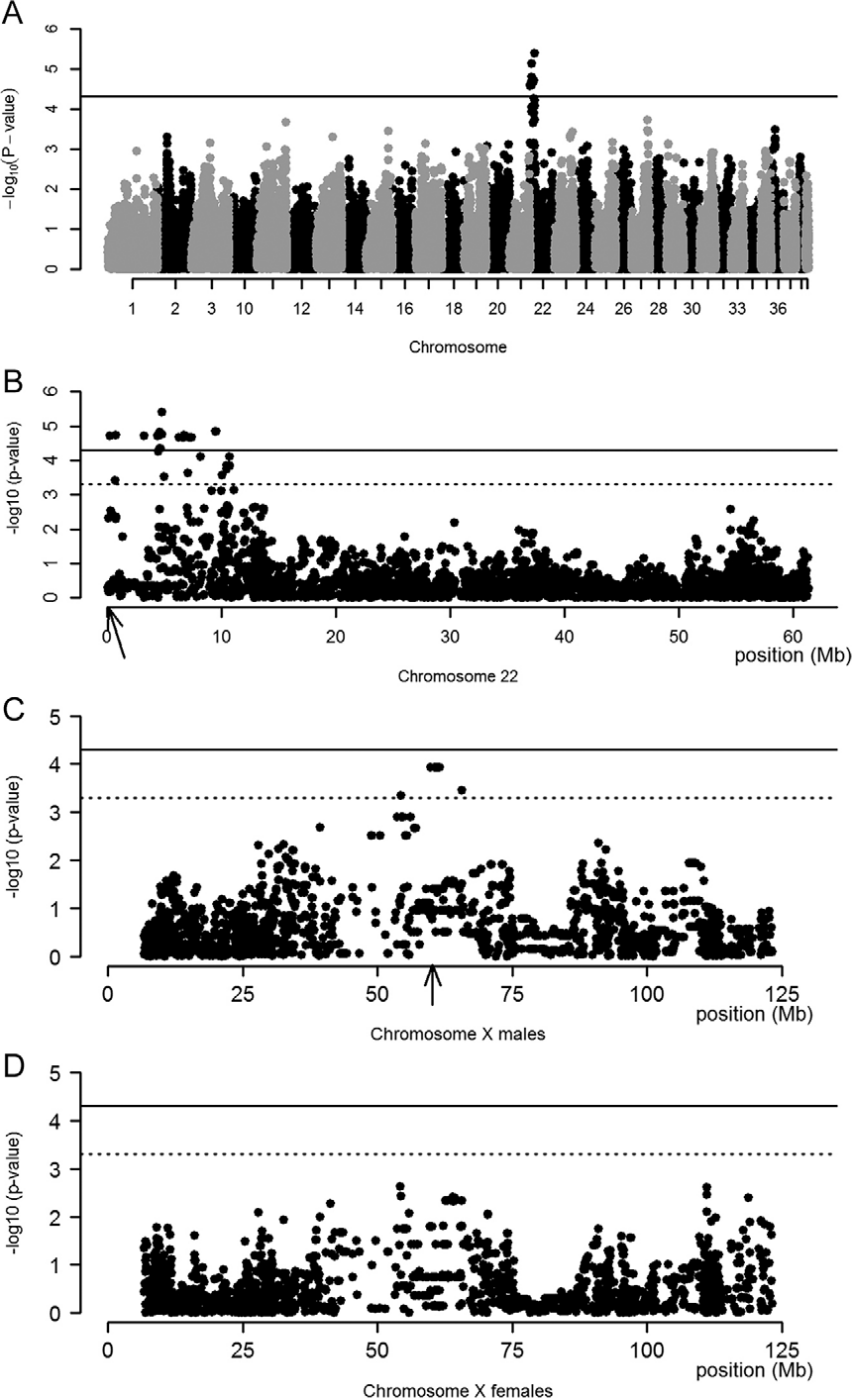
**Manhattan plots for hepatic copper score in Labrador retrievers.** (A) Manhattan plot of hepatic copper score in 235 Labrador retrievers shows a genome-wide association signal at chromosome 22. (B) Manhattan plot of chromosome 22 shows that the signal comprises an LD block comprising the first 10 Mb of the chromosome. The arrow indicates the location of *ATP7B*. (C) Mapping results for hepatic copper score at the X chromosome in male dogs shows an association signal at position X:60203319-60356690 (CanFam 3.1). The arrows indicate the location of *ATP7A*. (D) Mapping results for hepatic copper score at the X chromosome in females shows no substantial association. Note that the solid line indicates the boundary for suggestive genome-wide association at a significance level of *P*=5×10^−5^. The dotted line indicates the boundary used for determination of the crucial regions at *P*=5×10^−4^.

After quality control of genotype data used for the GWAS, 35.7% of SNPs were removed because of a low minor allele frequency and 0.7% of SNPs had a call rate of less than 98%, leaving 109,496 SNPs and 235 dogs for analysis. Population stratification was determined by a multidimensional scaling plot (Fig. S1), and inflation of the test statistic was inspected by a QQ-plot (Fig. S2). The heritability of hepatic copper score was estimated to be 0.48. The highest association signal using hepatic copper level as a quantitative trait in a linear mixed model was identified on chromosome 22, with a *P*-value of 2.4×10^−6^ and a β of 0.65 (s.e. 0.14; Fig. 2A). Inspection of the region showed a linkage disequilibrium (LD) block at the first 10 Mb of the chromosome. The crucial interval was determined to be at 22:152034-10753911, which contained 83 genes, including the candidate gene *ATP7B*, located at position 22:162769-225266 (Canfam 3.1; Fig. 2B).

A stratified analysis for males and females was performed (Fig. 2C,D). In the male subset, the crucial region was located at X:54322875-65713151 and contained 77 genes. The highest association signal [*P*=1.1×10^−4^ and a β of −0.57 (s.e. 0.15)] was caused by six SNPs that formed an LD block of 8.2 Mb, including the candidate gene *ATP7A* at position X:60203319-60356690 (Fig. 2C).

Given the fact that *ATP7A* and *ATP7B* code for copper transporters, they were strong candidate genes for involvement in copper toxicosis; therefore, we focused on these genes for in-depth DNA sequence analysis. A list of observed DNA variants and their effect on the hepatic copper score is presented in Table 1.

**Table 1.**
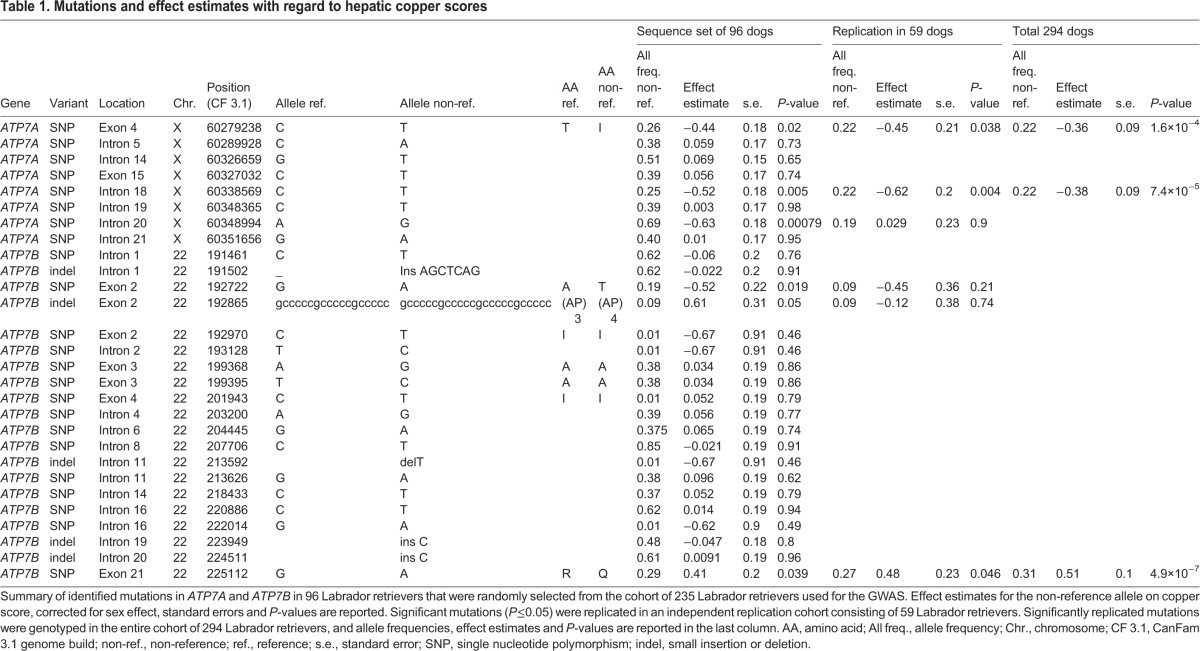
**Mutations and effect estimates with regard to hepatic copper scores**

The five nominally significant variations (*P*≤0.05) were genotyped in an independent cohort of 59 Labrador retrievers (Table 1; Table S1). The association with hepatic copper score was replicated for the non-synonymous nucleotide substitution of *ATP7A* at X-chromosomal position 60279238 (ENSCAFT00000049745 *ATP7A*:c.980C>T), the intronic variation in *ATP7A* (position X:60338569) and the non-synonymous nucleotide substitution of *ATP7B* at chromosome 22, position 225112 (ENSCAFT00000006859 *ATP7B*:c.4358G>A). When analysed in the complete data set (*n*=294), the *P*-values were similar to or lower than initially derived in the GWAS; 1.6×10^−4^ and 4.9×10^−7^ for *ATP7A*:c.980C>T and *ATP7B*:c.4358G>A, respectively (Table 1).

When inspecting the effect of the combined genotypes for *ATP7A* and *ATP7B* on hepatic histological copper levels (Table 2), we appreciated that for both sexes *ATP7B*:c.4358A was associated with increased hepatic copper levels in an additive way. This effect is most clear in female dogs, leading to a significant difference between copper levels of dogs homozygous for *ATP7B*:c.4358A and any of the other dogs. Concurrent presence of *ATP7A*:c.980T attenuated the copper accumulation effect in both sexes. This effect was most notable in males, leading to a significant difference between dogs with and without the mutant allele of *ATP7A* (Table 2).

**Table 2.**
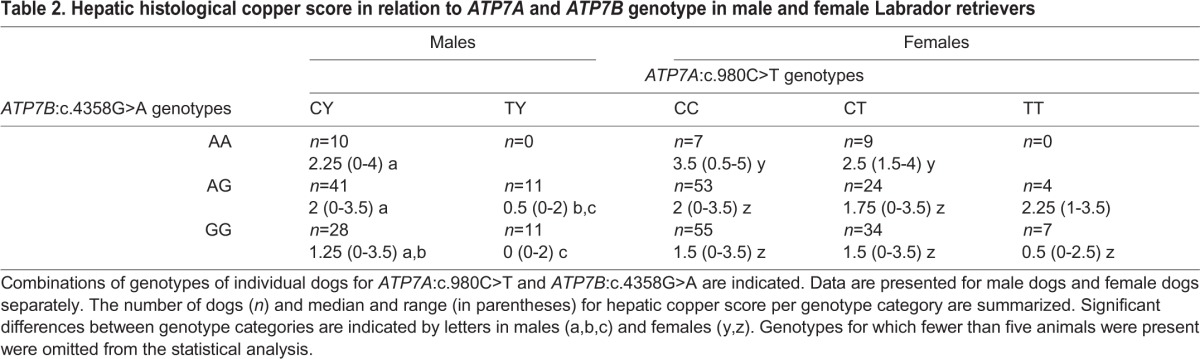
**Hepatic histological copper score in relation to** ***ATP7A*** **and** ***ATP7B*** **genotype in male and female Labrador retrievers**

The heritability for hepatic copper score in the model including the co-variates sex, *ATP7A*:c.980C>T and *ATP7B*:c.4358G>A was calculated to be 0.42. Thus, the explained genetic variability by the two non-synonymous mutations is [(0.48−0.42)/0.48] 12.5%, of which 4.3% could be attributed to *ATP7A*:c.980C>T and 8.2% could be attributed to *ATP7B*:c.4358G>A.

**Fig. 3.**
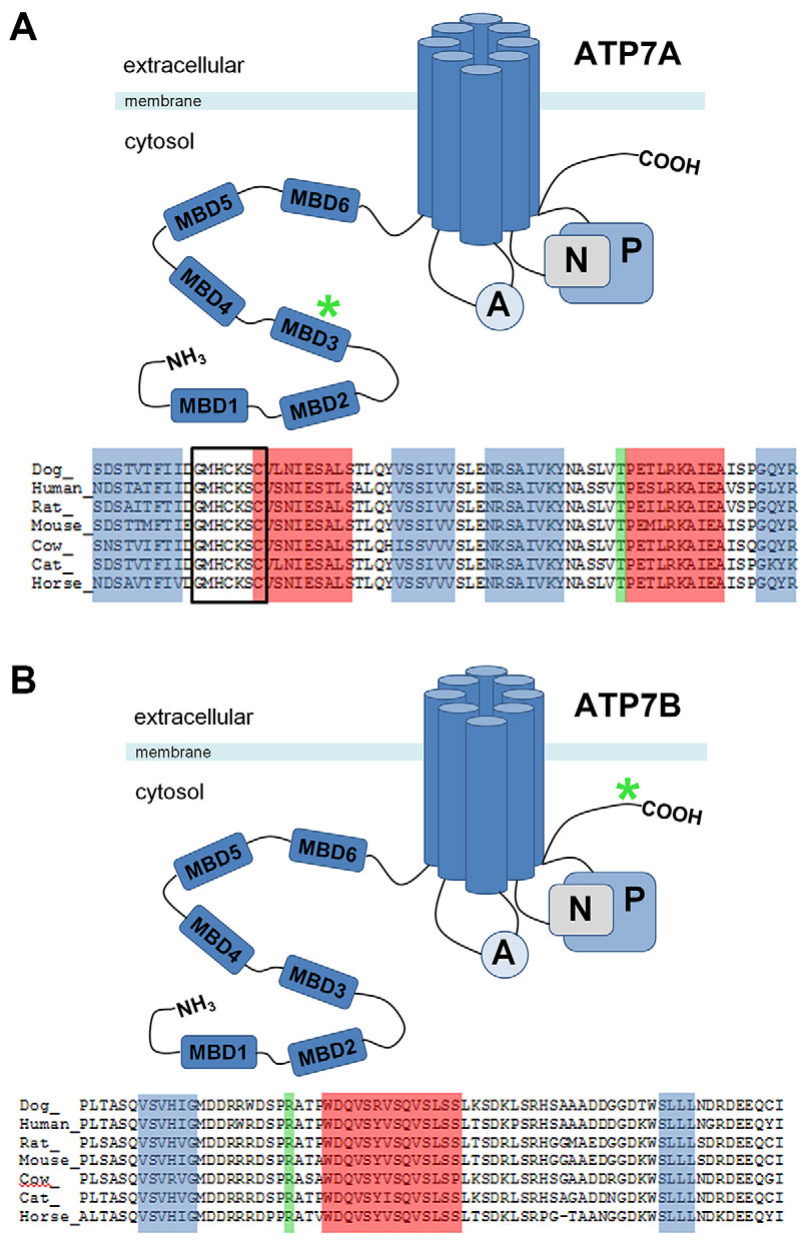
**Protein domains of ATP7A and ATP7B involved in copper toxicosis in Labrador retrievers.** Overview of the ATP7A (A) and ATP7B (B) proteins with the N-terminus, metal-binding domains (MBDs), actuator domain (A), nucleotide-binding domain (N), phosphorylation domain (P) and the C-terminus. The green asterisk indicates the position of the mutations. (A) Alignment of the region containing ATP7A^T327I^ (in green) shows a strong conservation of this amino acid position in the human, rat, mouse, cow, cat and horse. The copper-binding site XMXCXXC (boxed), predicted α-helix (red) and β-sheets (blue) are indicated. (B) Alignment of the region containing ATP7B^R1453Q^ (in green) shows a strong conservation of this amino acid position in the human, rat, mouse, cow, cat and horse. The predicted α-helix (red) and β-sheets (blue) are indicated.

*ATP7A*:c.980C>T resulted in an amino acid substitution from a threonine to an isoleucine in the third MBD of ATP7A at position 327 (Ensembl: ENSCAFT00000049745, ATP7A:p.Thr327Ile). *ATP7B*:c.4358G>A caused an amino acid substitution from an arginine to a glutamine in the C-terminus of the ATP7B protein at position 1453 (Ensembl: ENSCAFT00000006859, ATP7B:p.Arg1453Gln). Alignment of the amino acid sequence adjacent to canine ATP7A:p.Thr327Ile (ATP7A^T327I^) and ATP7B:p.Arg1453Gln (ATP7B^R1453Q^) indicated strong conservation of the domains in mammals (Fig. 3A,B).

### Functional evaluation of ATP7B^R1415Q^

#### ATP7B^R1415Q^ is partly mis-localized to the endoplasmic reticulum

**Fig. 4.**
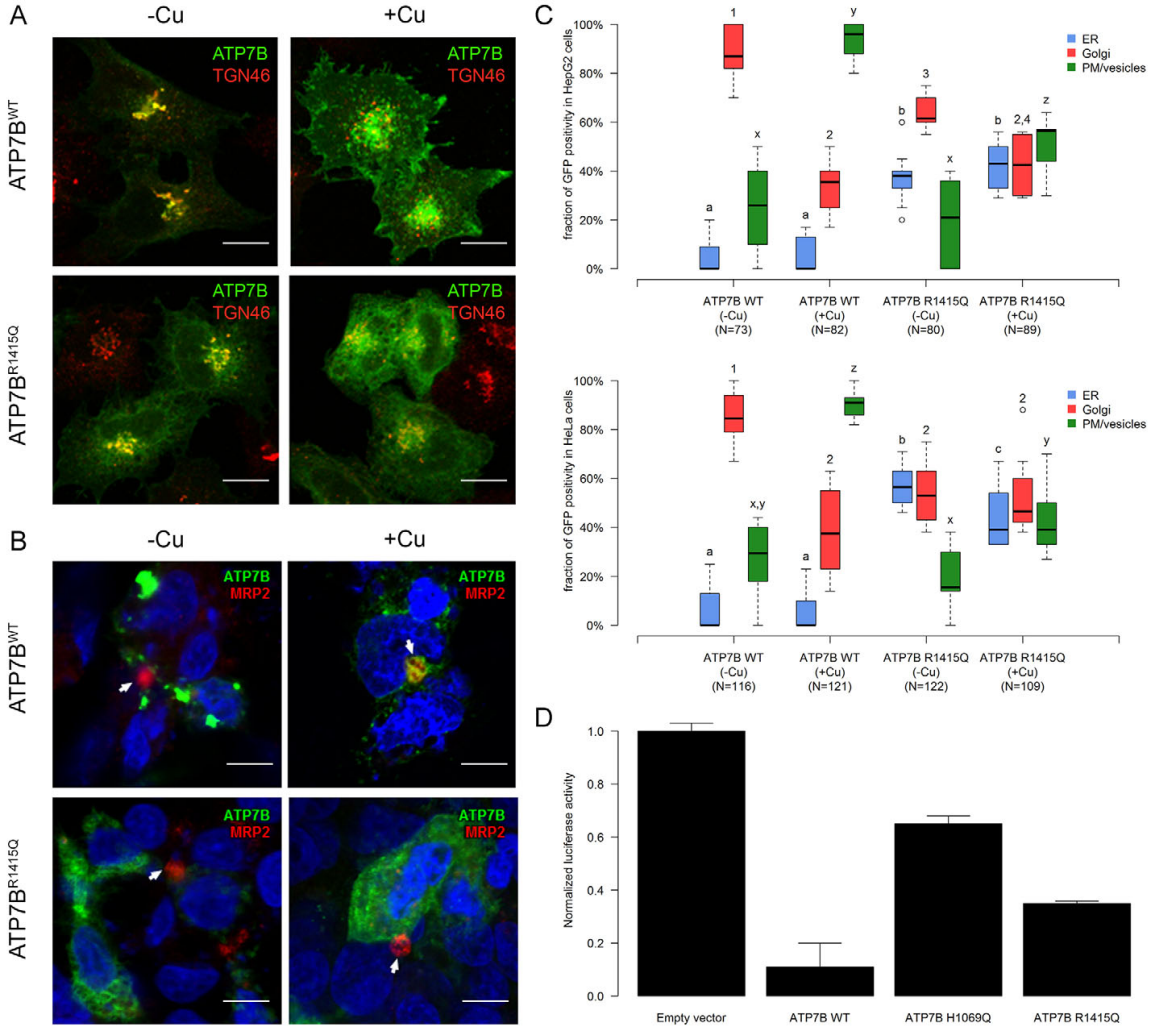
**Functional evaluation of ATP7B^R1415Q^.** (A) HeLa cells were transfected with either ATP7B^WT^ or ATP7B^R1415Q^ and either treated with 500 µM of the copper chelator BCS (Cu^−^) or incubated for 2 h with 200 µM CuSO_4_ (Cu^+^). In low-copper conditions, the localization of ATP7B^WT^ overlapped with the *trans*-Golgi marker TGN46, whereas copper stimulation induced ATP7B^WT^ trafficking to cytosolic vesicular structures and the cell surface (upper row). A significant cohort of ATP7B^R1415Q^ was detected in the endoplasmic reticulum network-like membranes in both low- and high-copper conditions (lower row). White scale bars: 5 µm. (B) Polarized HepG2 cells were exposed to BCS or CuSO_4_ for 4 h and stained with canalicular marker MRP2 and nuclear marker DAPI. White arrows indicate canalicular vacuole (cyst), which does not contain either wild-type or mutant ATP7B in chelator-treated cells. In contrast, the canalicular vacuole receives ATP7B^WT^ upon Cu stimulation, whereas ATP7B^R1415Q^ fails to reach the canalicular surface domain. White scale bars: 5 µm. (C) The intracellular distribution of GFP-tagged ATP7B^WT^ and ATP7B^R1415Q^ protein was analysed in HeLa (lower graph) and HepG2 (upper graph) cells in low- and high-copper circumstances. The number of cells used for quantification is indicated in parentheses. Significant differences (*P*<0.05) in the distribution of GFP within the cellular compartments are indicated with a,b,c (ER), 1,2,3 (Golgi) or x,y,z (plasma membrane or vesicles). There was an increase in the percentage of cells that exhibited GFP signal in the endoplasmic reticulum upon expression of ATP7B^R1415Q^-GFP, whereas the number of cells with ATP7B^WT^-GFP in the endoplasmic reticulum was very low in both HeLa and HepG2 cells. (D) HeLa cells were transfected with empty vector, ATP7B^WT^, ATP7B^H1069Q^ or ATP7B ^R1415Q^ and the metal-responsive element (MRE)-luciferase reporter for 24 h and treated with 200 μM CuSO_4_ for 24 h. Induction of the MRE-luciferase reporter was reduced when ATP7B^WT^ was co-expressed in the cells, whereas transfection of the partly active ATP7B^H1069Q^ mutant resulted in only modest inhibition of MRE-luciferase activation. Cells expressing ATP7B^R1415Q^ exhibited substantial reduction of reporter activity, indicating that ATP7B^R1415Q^ is still capable of transporting copper away from the cytosol. Bars represent the mean values of normalized luciferase activity (see Materials and Methods), and standard deviation is depicted by the error bars.

To analyse how the canine ATP7B:p.Arg1453Gln might affect copper homeostasis at the cellular level, we expressed the mutant protein exogenously in a cell line. To investigate the consequences of canine ATP7B:p.Arg1453Gln substitution on the subcellular localization of ATP7B, the conserved arginine (Arg) at position 1415 (Ensembl: ENST00000242839) of human GFP-tagged ATP7B was replaced by a glutamine (Gln). Both human ATP7B:p.Arg1415-GFP (ATP7B^WT^) and ATP7B:Gln1415-GFP (ATP7B^R1415Q^) were expressed in HeLa cells and and treated with either bathocuproine disulphonate (BCS) or 200 μM CuSO_4_. Confocal microscopy revealed that in low-Cu^2+^ conditions, ATP7B^WT^-GFP exhibited significant overlap with the *trans*-Golgi marker TGN46 as reported before (Barnes et al., 2009), whereas stimulation with CuSO_4_ induced ATP7B^WT^-GFP trafficking to the cell surface and numerous cytosolic vesicular structures (Fig. 4A; Fig. S3). In contrast, a significant amount of ATP7B^R1415Q^-GFP was detected in the endoplasmic reticulum (ER) network-like membranes in both low- and high-Cu^2+^ conditions (Fig. 4A; Fig. S3), although substantial amounts of the protein were delivered to the Golgi and post-Golgi compartments. Morphometric analysis confirmed an increase in the percentage of cells that exhibited GFP signal in the ER upon expression of ATP7B^R1415Q^, whereas the number of cells with ATP7B^WT^-GFP in the ER was very low (Fig. 4C). Partial mis-localization of ATP7B^R1415Q^ to the ER compartment was also observed in an HepG2 cell line (Fig. 4B,C). This indicated that the substitution of arginine to glutamine impairs (at least partly) appropriate compartmentalization of ATP7B.

#### ATP7B^R1415Q^ fails to reach the canalicular domain of polarized HepG2 cells

To verify whether ATP7B^R1415Q^ can be targeted to the apical (canalicular) domain of hepatic cells, which is necessary to remove excess Cu^2+^ from hepatocytes into bile, we grew HepG2 cells in the conditions that allow their maximal polarization (Slimane et al., 2003). Then, the cells were transfected with either ATP7B^WT^-GFP or ATP7B^R1415Q^-GFP and subsequently incubated with 200 μM CuSO_4_. Stimulation with copper resulted in delivery of ATP7B^WT^-GFP to the apical surface domains, which was labelled with the canalicular marker multidrug resistance protein 2 (MRP2) (Fig. 4B; Fig. S4). On the contrary, ATP7B^R1415Q^-GFP failed to arrive at the canalicular surface of the cells (Fig. 4B; Fig. S4) and remained mainly in the intracellular cytoplasmic structures, as it has been already demonstrated for another ER-retained mutant, ATP7B^H1069Q^ (Polishchuk et al., 2014). These results indicated that the mutant cannot be delivered efficiently to regular copper-excretion sites in polarized hepatocytes.

#### ATP7B^R1415Q^ exhibits copper-transporting activity

To assess the ability of ATP7B^R1415Q^ to transport copper, we transfected HeLa cells with the metal-responsive element (MRE)-luciferase reporter, a copper sensor based on the metallothionein-1 promoter that responds to bioavailable cytosolic copper and that was previously used to characterize cellular copper import (van den Berghe et al., 2007). Co-expression of MRE-luciferase reporter with empty GFP vector resulted in strong induction of luciferase activity (Fig. 4D) upon exposure of the cells to CuSO_4_. The induction of this copper-dependent reporter was significantly reduced when ATP7B^WT^ was co-expressed in the cells, whereas transfection of the partly active ATP7B^H1069Q^ mutant resulted in only modest inhibition of MRE-luciferase activation (Fig. 4D). Cells expressing ATP7B^R1415Q^ exhibited substantial reduction of reporter activity (Fig. 4D), while expressing a similar amount of protein to ATP7B^WT^-transfected cells (Fig. S5). This observation indicates that ATP7B^R1415Q^ is still capable of transporting copper out of the cytosol and that observed differences are not the result of differences in protein expression.

Taken together, our findings indicate that accumulation of copper upon ATP7B^R1415Q^ expression occurs mainly as a result of failure of the mutant to reach the appropriate copper-excretion compartments rather than as a result of a reduction in its copper-translocating activity.

#### Canine dermal fibroblasts expressing ATP7A^T327I^ accumulate more copper than wild-type fibroblasts

To ascertain whether ATP7A:9.Thr327Ile (ATP7AT^327I^) influenced cellular copper accumulation, a copper uptake and retention assay (Moller et al., 2011) was performed using copper 64 isotope (^64^Cu) in dermal fibroblasts.

Fibroblasts with ATP7A^WT^ were derived from five female and three male dogs, and fibroblasts with the variant ATP7A^T327I^ were derived from four female and three male dogs, and used for the ^64^Cu experiments. For the ^65^Zinc experiments, fibroblasts from three ATP7A^WT^ dogs (two females, one male) and three ATP7A^T327I^ dogs (one female, two males) were used. All dogs used for the fibroblast studies that were homo- or hemizygous for *ATP7A*:c.980C were homo- or hemizygous for the reference allele for the SNP in intron 18 (position 60338569), and all dogs that were homo- or hemizygous for *ATP7A*:c.980T were homo- or hemizygous for the non-reference allele.

**Fig. 5.**
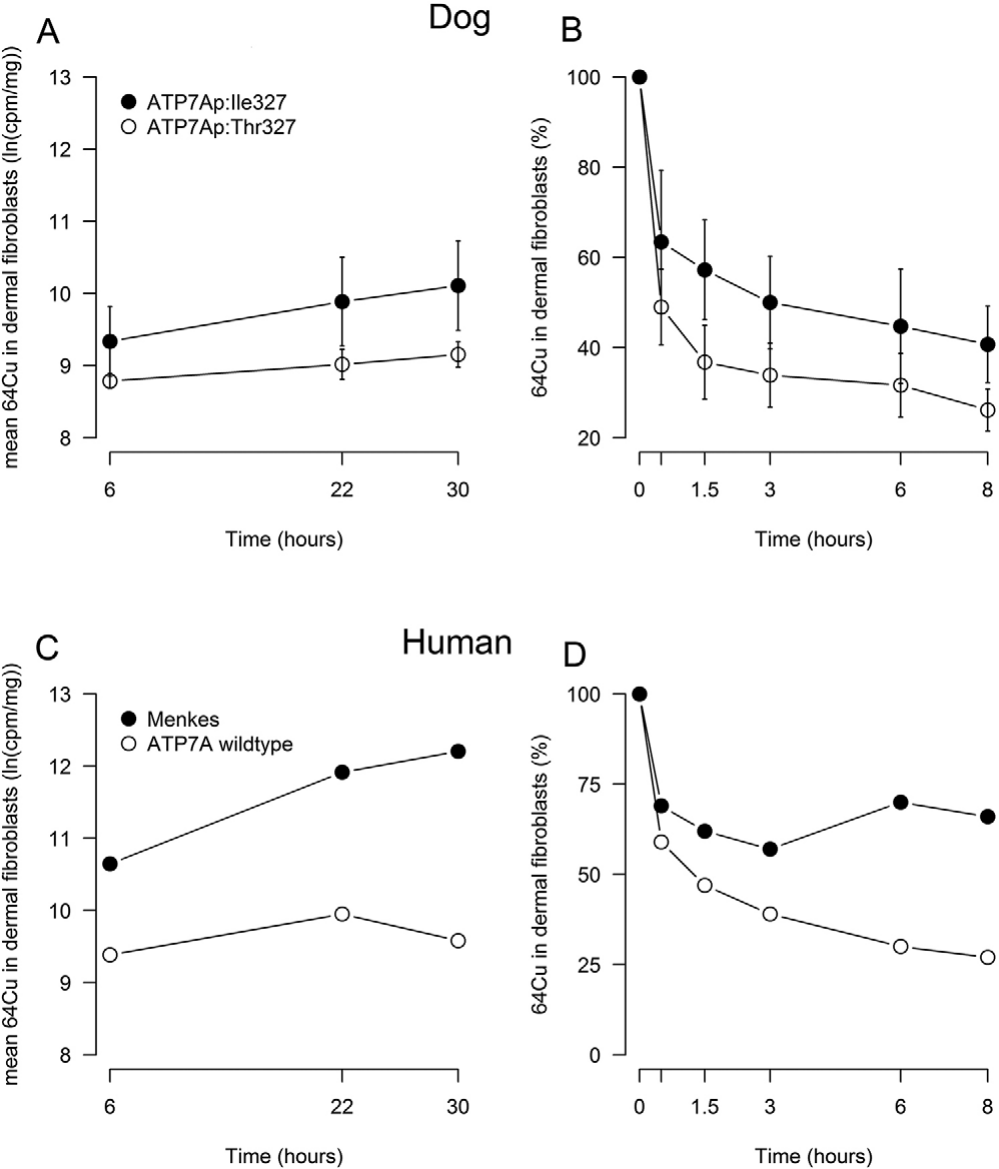
**Copper accumulation and retention in canine dermal fibroblasts.** (A) Copper influx in canine fibroblasts. Dermal fibroblasts derived from ATP7A:p.Ile327 (ATP7A^T327I^) dogs showed significantly more uptake of ^64^Cu than dermal fibroblasts derived from dogs with ATP7A:p.Thr327 (ATP7A^WT^). The overall difference in copper accumulation was 86% (95% confidence interval 25-176%, *P*=6.1×10^−3^). The overall increase in ^64^Cu was compared to time point 6 h. After 22 h, the estimated increase in copper was 45% (95% confidence interval 27-64%, *P*<1.0×10^−4^), and after 30 h this was 72% (95% confidence interval 45-104%, *P*<1.0×10^−4^). Dots represent mean values, and standard deviation is represented by the error bars. (B) Copper efflux in canine fibroblasts. Dermal fibroblasts from ATP7A^T327I^ dogs showed significantly more retention of copper than dermal fibroblasts derived from dogs with ATP7A^WT^. The overall average export rate in ATP7A^T327I^ was significantly lower than in ATP7A^WT^ (*P*=0.011). Dots represent mean percentages, and standard deviation is represented by the error bars. (C) Copper influx in human fibroblasts. Dermal fibroblasts from a human with Menkes disease showed considerably more uptake of copper than dermal fibroblasts derived from a human with ATP7A wild type. (D) Copper efflux in human fibroblasts. Dermal fibroblasts from a human with Menkes disease showed considerably more retention of copper than dermal fibroblasts derived from a person with ATP7A wild type.

The model for ^64^Cu accumulation in fibroblasts that included the fixed factors time and mutation had the lowest Akaike's information criterion (AIC). Overall, a significant increase of copper at time points 22 and 30 h was present. The fibroblasts derived from the ATP7A^T327I^ dogs accumulated significantly more copper than fibroblasts derived from ATP7A^WT^ dogs (Fig. 5A).

To establish whether the differences in ^64^Cu levels between fibroblasts were the result of abrogated efflux, a chase method was used, after preloading of cells with copper for 22 h. The average export rate of fibroblasts with the ATP7A^T327I^ variant was significantly lower compared with the average export rate in fibroblasts derived from ATP7A^WT^ (Fig. 5B). Western blots of dermal fibroblasts derived from ATP7A^WT^ and ATP7A^T327I^ dogs did not show differences in ATP7A protein expression (Fig. S6).

As a control, fibroblasts from a human fibroblast cell line (ATP7A^WT^) and a human Menkes disease-affected individual (Menkes; both *n*=1) were included in the experiments. Fibroblasts derived from the human with Menkes disease accumulated considerably more ^64^Cu than the ATP7A^WT^ fibroblasts (Fig. 5C) and showed abrogated efflux (Fig. 5D).

Handling of an alternative trace metal in canine fibroblasts was not influenced by the *ATP7A* genotype. Incubation of fibroblasts with ^65^Zn resulted in similar patterns of accumulation of isotope over time (Fig. S7A), and the release kinetics of newly accumulated ^65^Zn were comparable between the fibroblasts derived from ATP7A^WT^ dogs and dogs with ATP7A^T327I^ (Fig. S7B). In summary, this experiment showed that fibroblasts derived from dogs with the ATP7A^T327I^ accumulate more copper than fibroblasts derived from dogs with ATP7A^WT^ and that copper accumulation might be the result of decreased copper excretion by ATP7A^T327I^ fibroblasts.

#### Copper-induced trafficking is not changed in cells expressing ATP7A^T327I^

To investigate whether the observed decrease in copper excretion was a result of aberrant trafficking, immunofluorescence studies were performed in canine dermal fibroblasts derived from ATP7A^WT^ and ATP7A^T327I^ dogs. Both variants reside in the Golgi in low-copper circumstances. After 2 h incubation with copper, both ATP7A^WT^ and ATP7A^T327I^ exhibited substantial redistribution from the Golgi to the small post-Golgi vesicles and plasma membrane (Fig. S8).

To understand whether basolateral targeting of ATP7A^T327I^ was compromised, apicobasal distribution of both ATP7A^WT^ and ATP7A^T327I^ was investigated in filter-grown polarized Madin–Darby canine kidney (MDCK) cells. The MDCK cells were transfected with GFP-tagged versions of either ATP7A^WT^ or ATP7A^T327I^ and exposed to CuSO_4_ for 4 h. Confocal microscopy revealed efficient delivery of ATP7A^WT^ to the lateral cell surface domain of MDCK located below the occludin-labelled belt of tight junctions. As did the wild-type protein, ATP7A^T327I^ exhibited efficient targeting to the lateral walls of MDCK cells (Fig. S9), indicating that its polarized delivery in epithelial cells remained unaffected.

## DISCUSSION

We performed a GWAS in 235 Labrador retrievers to identify causal genes for copper toxicosis. We identified two chromosomal loci that were positively and negatively associated with hepatic copper levels. The two loci respectively harboured the genes coding for the copper transporters ATP7B and ATP7A. Subsequent Sanger sequencing in 96 Labrador retrievers and validation of mutations in an independent cohort of 59 Labrador retrievers identified a non-synonymous SNP in *ATP7B* associated with an increase in hepatic copper levels, and a non-synonymous and intronic SNP in *ATP7A* associated with a decrease in hepatic copper levels*.* Functional follow-up studies for the non-synonymous mutations provided biological evidence for their involvement in a disturbed copper metabolism.

Copper toxicosis in Labrador retrievers and Wilson disease are similar with regard to the amount of hepatic copper accumulation, presence of iron accumulation and progression to liver cirrhosis. Electron microscopy of liver sections of affected Labrador retrievers revealed ultrastructural changes that are similar to those observed in humans with Wilson disease. We show, for the first time, that Labrador retrievers and humans with Wilson disease share *ATP7B* as the causal gene. However, histopathological differences between copper toxicosis in Labrador retrievers and Wilson disease are present and include predominant centrolobular copper accumulation in Labrador retrievers versus a periportal distribution in humans with Wilson disease. Furthermore, fatty degeneration and Mallory bodies are not recognized in liver histopathology from Labrador retrievers. In both diseases, the age of onset is usually in middle or older age, but neurological symptoms and Kaiser–Fleisher rings have not been recognized in Labrador retrievers.

Dietary intake of copper seems important in disease progression in Labrador retrievers (Fieten et al., 2014, 2012a) and, in this regard, Labrador retriever copper toxicosis is comparable to endemic Tyrolean infantile cirrhosis and Indian childhood cirrhosis. The observed differences among copper toxicosis diseases in humans and Labrador retrievers are intriguing, and further studies are needed to identify whether they can be attributed to general species differences in copper metabolism or to other, yet to be identified gene mutations in either species.

ATP7B is highly expressed in hepatocytes, where it is located in the *trans*-Golgi network in low-copper conditions. With increasing intracellular copper levels, ATP7B traffics to endo-lysosomal vesicular structures and to the canalicular surface of hepatocytes (Guo et al., 2005; Polishchuk et al., 2014; Schaefer et al., 1999) to facilitate copper excretion into the bile. The C-terminus has an important role in regulating copper-responsive trafficking. Mutations of residues in this region are suggested to contribute to aberrant Golgi retention of ATP7B in high-copper circumstances (Braiterman et al., 2011). Our fluorescence studies did not show a significant difference in GFP signals in both HeLa and polarized HepG2 cells between the wild type and ATP7B^R1415Q^ after copper treatment. However, ATP7B^R1415Q^ showed increased retention in the ER and failure to reach the canalicular surface in polarized hepatic cells in high-copper conditions, which might occur because of mis-folding of the protein. Mis-folding and aberrant retention in the ER have been previously reported in Wilson disease-associated *ATP7B* mutations (Gupta et al., 2011; van den Berghe et al., 2009). The luciferase assay demonstrated that the copper-transport capacity of ATP7B^R1415Q^ showed only a modest impairment, indicating that the functional effect of ATP7B^R1415Q^ in hepatic copper accumulation in Labrador retrievers results from mis-localization of the protein, rather than from severely decreased copper-transport capacity.

In Labrador retrievers, there is a strong female predisposition for disease (Fieten et al., 2012b). X-chromosomal mapping in a combined data set can be hampered by hemizygosity in males, and thereby, possibly cause disturbance of association signals. Therefore, we analysed males and females separately. In the region harbouring *ATP7A*, we identified a suggestive signal of association with lower hepatic copper levels in male dogs. We hypothesized that mutations in *ATP7A* might modify copper accumulation, and this gene was therefore analysed by DNA sequence analysis. The identified missense mutation *ATP7A:*c.980C>T results in the amino acid substitution ATP7A:p.Thr327Ile in MBD3 of ATP7A and was negatively associated with hepatic copper levels. MBD3 is located in the cytoplasm and has the ability to receive copper from the Atox1 chaperone (Strausak et al., 2003). MBD3 of ATP7A is only metallated in elevated copper conditions (Banci et al., 2010) and is thought to be an important domain for regulating ATP7A activity via cell-signalling pathways (Veldhuis et al., 2011). It has been shown that ATP7A:p.T327 is an important phosphorylation site of ATP7A (Veldhuis et al., 2009).

To investigate whether ATP7A^T327I^ influenced cellular copper accumulation, we studied copper uptake and retention in dermal fibroblasts derived from Labrador retrievers hemi- or homozygous for either ATP7A^WT^ or ATP7A^T327I^. Mutant fibroblasts accumulated significantly more copper and showed a decreased efflux rate without a change in protein expression levels, indicating a functional impairment of ATP7A. We also investigated whether this could be a result of aberrant trafficking or sorting of the mutant upon copper stimulation. Hereto, we used wild-type and ATP7A^T327I^ dermal fibroblasts and polarized MDCKs transfected with either ATP7A^WT^ or ATP7A^T327I^. In both experiments, correct trafficking to the plasma membrane and delivery to the basolateral membrane (in polarized MDCKs) upon copper stimulation was observed. These results are in line with previous observations that the phosphorylation site ATP7A:pT327 is not involved in aberrant trafficking of ATP7A (Veldhuis et al., 2009), but is rather predicted to stabilize a conformational change in ATP7A, exposing the CxxC copper-binding region of MBD3, which might affect catalytic activity of ATP7A (Veldhuis et al., 2011). This might indicate that catalytic activity, rather than aberrant trafficking of ATP7A, is responsible for the observed decrease in hepatic copper levels in Labrador retrievers.

In humans, disease-causing mutations in the third metal-binding domain of ATP7A were not reported (Kaler, 2011). For ATP7B, 15 (three silent, 12 Wilson disease-causing) variants in MBD3 are described. Two mutations, c.915T>A (Loudianos et al., 1998) and c.918-931del (Davies et al., 2008), lead to a premature stop and a concomitant absence of ATP7B:p.Ser306 (corresponding to ATP7A:p.Thr327). Both of these mutations cause Wilson disease.

Another single base-pair substitution, C>T, located in intron 18 (position 60338569, CF 3.1), was also inversely related to hepatic copper score. In the dogs from which the fibroblasts were derived, the SNP in intron 18 and *ATP7A:*c.980C>T were in complete LD, and therefore we cannot rule out the possibility that the intronic variation exerts an effect on *ATP7A* gene function; however, we consider it less likely. The alleles are both pyrimidines within a stretch of pyrimidines, are not situated close to an intron-exon junction, and the mutation is not expected to activate a cryptic splice site. In the total data set, the intronic SNP was in high LD (*r*^2^ 0.92, D′ 0.97) with *ATP7A:*c.980C>T, making it likely that the observed association is a result of the fact that this SNP is in LD with *ATP7A:*c.980C>T.

*ATP7B*:c.4358G>A showed an additive effect on hepatic copper levels, which was most significant in female dogs (Fig. 2). Copper-accumulating effects of *ATP7B*:c.4358G>A were modified by the presence of *ATP7A:*c.980C>T, and this effect was most clear in male dogs (Table 2). In accordance with observations in humans with Menkes disease, where aberrant *ATP7A* function results in an impaired intestinal copper uptake, we suggest that in Labrador retrievers ATP7A^T327I^ might result in decreased intestinal copper uptake, thereby attenuating hepatic copper accumulation because of ATP7B^R1453Q^. Other possible mechanisms influencing copper homeostasis in Labrador retrievers and, more specifically, the interplay between ATP7A and ATP7B might include regulation by hormones, inflammatory cytokines, growth factors or a yet unidentified small molecule acting as a regulator of systemic copper metabolism (Kim et al., 2010).

In humans with Wilson disease, a wide variation in clinical symptoms and hepatic copper accumulation is noticed, which is currently unexplained (de Bie et al., 2007; Lee et al., 2011; Riordan and Williams, 2001; Shah et al., 1997). When we translate our observations in the Labrador retrievers to the human disease, we might think of *ATP7A* as a possible modifier gene in Wilson disease. Variations in *ATP7A* with a small effect on protein function might reduce the rate of copper accumulation in humans with Wilson disease, thereby modifying the disease phenotype, for example by delaying the age at onset of clinical symptoms. In fact, part of the apparent discrepancy between the genetic incidence of Wilson disease and the number of affected individuals diagnosed (Coffey et al., 2013) might be a result of a slower rate of copper accumulation that does not reach toxic levels in the life span of some individuals. In a recent pilot study, two SNPs in *ATP7A* were genotyped in humans with Wilson disease. No association was identified between the *ATP7A* polymorphisms and the presence of Wilson disease or clinical phenotypic parameters (Przybyłkowski et al., 2014). However, these results clearly do not exclude *ATP7A* as a candidate modifier, because it was a very limited investigation of *ATP7A* and a small data set.

In conclusion, the present study illustrates the potential of canine inbred populations for the unravelling of complex hereditary diseases. We identified the involvement of the copper transporter ATP7B in copper toxicosis in the Labrador retriever, identifying this breed as the first naturally occurring non-rodent mammalian model for Wilson disease. The concurrent identification of a functional mutation in *ATP7A* suggests a role for the Menkes gene as a disease modifier in copper-metabolism disorders. Owing to its long life span and large body size, the Labrador retriever will be an invaluable large-animal model for the development of new therapies, including gene therapy, beneficial to both human and canine affected individuals.

## MATERIALS AND METHODS

### Dogs

Samples for the initial GWAS and DNA sequencing study were collected between 2003 and 2009, and samples for the replication cohort were collected between 2009 and 2013 at the Faculty of Veterinary Medicine, Utrecht University, with informed consent of the owners. The procedures were approved by the Utrecht University Ethical Committee as required under Dutch legislation. In addition, procedures were reviewed and approved by the WALTHAM Centre for Pet Nutrition. Each dog underwent a physical examination. An EDTA blood sample of 4 ml was used for DNA extraction from peripheral white blood cells by a salt extraction procedure (Miller et al., 1988). For diagnostic purposes, liver biopsies were obtained by the Menghini technique, ultrasound guided with a Tru-cut device and 14 gauge needle, during laparoscopy or laparotomy (Rothuizen et al., 2006). Local anaesthetics (infiltration of the skin and subcutaneous tissues with lidocaine) were applied for Tru-cut liver biopsies. When necessary, dogs were sedated with propofol (dosage to effect) during the liver biopsy procedure. For liver biopsies obtained by laparoscopy or laparotomy, dogs underwent general anaesthesia. The anaesthesia protocols used were individually designed based on the anaesthetic risk classification of the American Association of Anesthesiologists. Tissue specimens were embedded in paraffin, and 4 µm thick sections were mounted on glass slides. Sections were stained with haematoxylin and eosin, rubeanic acid (Uzman, 1956) for copper and reticulin (Gordon and Sweets, 1936). All samples were evaluated and graded by one board-certified pathologist (T.S.G.A.M.v.d.I.) according to World Small Animal Veterinary Association standards (Van Den Ingh et al., 2006). Hepatic copper accumulation was histologically graded on a scale from zero to five as described previously (Fieten and Rothuizen, 2014).

### Electron microscopy

For routine electron microscopic analysis, liver specimens were obtained from two Labrador retrievers affected with copper toxicosis and one healthy dog of mixed breed. Biopsies were fixed in 1% glutaraldehyde in 0.2 M HEPES buffer. Small blocks of liver tissue were post-fixed in uranyl acetate and in OsO_4_. After dehydration through a graded series of ethanol, the tissue samples were cleared in propylene oxide, embedded in the Epoxy resin (Epon 812) and polymerized at 60°C for 72 h. From each sample, thin sections were cut with a Leica EM UC7 ultra-microtome. Thin sections were investigated further using a FEI Tecnai-12 (FEI; Eindhoven, The Netherlands) electron microscope equipped with a Veletta CCD camera for digital image acquisition.

### Genome-wide association study

DNA samples from 235 Labrador retrievers were genotyped using Illumina's Canine HD BeadChip, containing ∼170,000 SNPs. Data were analysed in the R-package (R Development Core Team, 2011) ‘GenABEL’ version 1.6-6 (Aulchenko et al., 2007). Quality-control thresholds were set using the function ‘check.marker’. A threshold of 98% successful genotypes per individual and 98% successful genotypes per SNP was used. SNPs for which at least 20 heterozygotes were typed were included in the analysis. Furthermore, a check for sex mismatch (based on genotypes at the X chromosome), duplicate sample checks and checks for heterozygosity outliers were performed. The phenotype copper score was analysed as a quantitative trait, and sex was modelled as a fixed factor. Stratification and cryptic relatedness were adjusted for by mixed models implemented in the function ‘mmscore’ (Chen and Abecasis, 2007). A correction for residual genomic control was applied. The heritability of hepatic copper score with correction for sex was calculated based on genotype kinship matrix with the function ‘polygenic_hglm’ based on the packages ‘hglm’ (Ronnegard et al., 2010) and ‘GenABEL’. The autosomes and pseudo-autosomal region of the X chromosome were analysed in male and female dogs together. An additional stratified analysis for males and females was performed to facilitate inspection of the X chromosome.

Genome-wide suggestive association criteria were set to an uncorrected *P*-value of *P*≤5×10^−5^. Boundaries for the crucial intervals were determined for a significance level of *P*≤5×10^−4^. Crucial intervals were screened for the presence of possible candidate genes.

### DNA sequence analysis and validation

Exons and intron-exon boundaries from positional candidate genes were selected for Sanger sequencing in 96 individual dogs randomly selected from the 235 Labrador retrievers used in the initial GWAS. Variants that were significantly associated with hepatic copper level were genotyped in an independent replication cohort of 59 Labrador retrievers and in all 294 dogs. Statistical analysis was performed with R version 2.11 (R Development Core Team, 2011). A linear regression model with sex as a covariate was used to test the association of the mutations identified with Sanger sequencing in the candidate genes with hepatic copper score. The mutations were modelled in an additive way.

Genotypes for the mutations that were significant after statistical testing in the replication cohort were genotyped by Sanger sequencing in the total cohort of 294 dogs. Differences in hepatic copper scores with respect to combined *ATP7A* and *ATP7B* genotype were determined using a Wilcoxon rank-sum test within males and females separately.

Genotypes for *ATP7B*:c.4358G>A and *ATP7A*:c.980C>T were added as independent covariates, and heritability was calculated by using the function ‘polygenic_hglm’ (Ronnegard et al., 2010) in GenABEL. The explained genetic variability was calculated by the following formula: (heritability of model without mutations as a covariate minus heritability including mutations as covariates/heritability without mutations as a covariate). In all heritability calculations, sex was added as a covariate.

### Functional evaluation of ATP7B^R1453Q^

Alignment of human and canine ATP7B protein sequence confirmed that the Arg at position 1453 in the canine protein corresponds to the Arg at position 1415 in the human ATP7B. The human pAdlox-ATP7B-GFP construct was used, and site-directed mutagenesis was performed to make pAdloxATP7B-p:Arg1415Gln-GFP. Both constructs were used for cell transfection and subsequent immunofluorescence and luciferase assays.

#### DNAs and site-directed mutagenesis

To prepare ATP7B:p.Arg1415Gln, the pAdlox-ATP7B-GFP construct (provided by S. Lutsenko, Baltimore, MD, USA) was used as the template, and site-directed mutagenesis was performed according to the manufacturer's instructions for point mutagenesis using the QuikChange II XL site-directed mutagenesis kit (Stratagene, La Jolla, CA, USA), using the following oligonucleotides: 5′-GGGACTCCCCCCAGGCCACACCATGGGACC-3′ and 5′-GGTCCCATGGTGTGGCCTGGGGGGAGTCCC-3′.

Given that *ATP7A* sequences are unstable in high-copy plasmids (reference PMID: 9571140), we used commercial DNA synthesis to generate silent mutations in the mouse *Atp7a* open reading frame in the hope that such changes would permit stable propagation in high-copy plasmids. Overlapping PCR was used to generate more than 1000 silent mutations within the mouse *Atp7a* open reading frame (Life Technologies, Grand Island, NY). Two synthetic DNA fragments were generated. The first was a 2.0 kb fragment flanked by *Acc*III/*Xba*I restriction sites, and the second was a 1.2 kb fragment flanked by *Xba*I/*Xho*I restriction sites. Each was subcloned in the high-copy pMK-RQ vector, and at least three clones were sequenced and found to lack new mutations, indicating that both fragments were stably propagated in *Escherichia coli*. A third DNA fragment that encompassed the last 1.4 kb of the native mouse *Atp7a* open reading frame was generated by PCR using primers 5′-ACCATCTAGACTCGAGATGGCTCATAAGGTAAAGGTAGTGGTATTTGATAAGACTGG and 5′-AGATGCGGCCGCTTACAGTGTGGTGTCATCATCTTCCCGGAAGTCG. A complete *Atp7a* construct was then generated by ligations involving this PCR product and both synthetic fragments, and subcloned into the expression construct, pQCXIP (Clontech). The resulting Atp7a expression plasmid was found to be stably propagated in *E. coli* and verified by sequence analysis. A PCR fragment containing the EGFP sequence flanked by *Mfe*I sites was appended to the 5′ region of Atp7a to generate the pAtp7a-GFP plasmid. GFP-tagged ATP7A^T327I^ mutant was generated using standard mutagenesis service by GenScript.

#### Reagents

Antibodies were obtained from the following sources: anti-ATP7A (Hycult Biotech, Plymouth Meeting, PA, USA), anti-Human Golgin-97 Mouse Monoclonal CDF4 (Molecular Probes, Paisley, UK), anti-GFP and anti-VAP-A from M.A. De Matteis (TIGEM, Naples, Italy), anti-α-tubulin (Sigma-Aldrich, St Louis, MO, USA), anti-TGN46 (AbD Serotec, Oxford, UK); anti-Na^+^/K^+^-ATPase (Abcam, Cambridge, UK), anti-MRP2 (Enzo Life Sciences, Lausanne, Switzerland), anti-occludin and secondary Alexa Fluor 568-conjugated antibodies (Invitrogen-Life Technologies, Grand Island, NY, USA). Dual-Luciferase^®^ reporter assay kit was from Promega (Madison, WI, USA).

#### Cells and transfection

Hela and HepG2 cells were routinely grown at 37°C in Dulbecco's modified Eagle's medium (DMEM), containing 10% FBS (fetal bovine serum with inactivated complement for HepG2) on coverslips and transfected with pAdlox-ATP7B-GFP, pAdlox-ATP7B-H1069Q-GFP and pAdlox-ATP7B-R1415Q-GFP using *Trans*IT^®^-LT1 and jetPEI^®^ polyplus transfection protocols for Hela and HepG2, respectively, according to the manufacturer's instructions. ATP7B-H1069Q was used as a control because it is one of the most common Wilson disease mutations in the Caucasian population and impairs ATP7B function. To reach polarization, HepG2 cells were grown according to the published protocol for 72 h (Slimane et al., 2003).

MDCK cells were grown on Transwell polycarbonate filters (Corning Costar, Lowell, MA, USA) in DMEM containing 10% FBS for 4-5 days to achieve sufficient polarization. GFP-tagged ATP7A-WT or ATP7B-T327I were transfected using *Trans*IT^®^-LT1 2 days before the experiment.

#### Luciferase assay

For the luciferase assay, the pAdloxATP7B-H1069Q-GFP construct was used as a positive control, because ATP7B-H1069Q is the most common mutation causing Wilson disease in the Caucasian population and proved to be functionally active. HeLa cells were plated in a 12-well plate and transfected with pGL3-E1b-TATA-4MRE reporter (van den Berghe et al., 2007; provided by Bart van de Sluis, Groningen, The Netherlands) and then co-transfected with either empty vector (pEGFPc1) or pAdloxATP7B-GFP or pAdloxATP7B-H1069Q-GFP or pAdloxATP7B-p:Arg1415Gln-GFP expression plasmids using the *Trans*IT^®^-LT1 transfection protocol. After 24 h, cells were treated with 200 μM CuSO_4_ for 24 h. Cells were subsequently harvested in a Passive Lysis Buffer (Promega, Madison, WI, USA) according to the manufacturer's instructions. Firefly luciferase and *Renilla* luciferase activities were measured with a Dual-Luciferase^®^ reporter assay system (Promega) on a GloMax^®^ 96 Microplate Luminometer (Promega) according to the manufacturer's instructions. Relative light units were calculated by dividing firefly measurements by *Renilla* measurements. All values were normalized to the ATP7B protein expression levels determined by ImageJ (Collins, 2007) analysis of western blot in the respective specimens. Western blot analysis was performed on lysates obtained by transfection of the cells with pEGFP-C1, pAdlox-ATP7B-GFP, pAdlox-ATP7B-H1069Q-GFP and pAdlox-ATP7B-:Arg1415Gln-GFP expression plasmids. Lysates were prepared via the addition of lysis buffer [0.5% Triton X-100, 20 mM Tris-HCl (pH 7.4), 150 mM NaCl, 1 mM EDTA (pH 8), 0.5% Np-40, 10% glycerol and 1× protease inhibitor cocktail (Sigma S88201)] for 10 min at room temperature. The mixture was placed into a microfuge tube, kept on ice for 10 min, and then spun at 17,000 ***g*** for 15 min at 4°C in a microcentrifuge. The quality of transfer was verified by Ponceau staining, and molecular weights were inferred by comparison to prestained markers (New England Biolabs, Ipswich, MA, USA).

### Functional evaluation of ATP7A^T327I^

Dermal fibroblast cells were derived from Labrador retrievers selected for the presence or absence of an *ATP7A*:c.980C>T substitution resulting in the ATP7A^T327I^ amino acid substitution in homozygous or hemizygous form. The human dermal fibroblast cells HB156 from an individual with clinical Menkes disease (cell line: DD0355, no. 91071704) were purchased from the European Collection of Cell Cultures (ECACC). ECACC is managed as part of the Health and Safety Executive (HSE) in the UK. The human HDFn dermal fibroblast line (Invitrogen, Bleiswijk, The Netherlands) was used as a control cell line.

Fibroblast cultures were routinely maintained in DMEM (Invitrogen) supplemented with l-glutamine (2 mM), 10% FBS, MEM non-essential amino acids (100 µM) and penicillin-streptomycin at 37°C in a humidified atmosphere of 5% CO_2_ in air. For experiments, canine cells were used between passage 4 and 9.

#### Radioisotope experiments

Methods for radioisotope experiments were based on those described previously (Spee et al., 2007). In short, ^64^Cu was prepared using metallic copper wire (1 mg) irradiated overnight to provide an induced activity of approximately 16.6 Ci/g (Reactor Institute, Delft, The Netherlands). Four hours after irradiation and 30 min before the start of the study, the copper wire was dissolved in 100 µl 10.3 M HNO_3_ and subsequently neutralized with 0.5 M NaOH. DMEM was added to give a final concentration of 10 mM copper. Fibroblasts were cultured from skin-punch biopsies, obtained with local anaesthesia (lidocaine infiltration).

Fibroblast cells were plated in triplicate in six-well plates in DMEM+10% FBS. Cells were incubated with 3 µM ^64^Cu for 6, 22 or 30 h. Culture medium was removed after treatment, and the cells were washed four times with Hanks solution containing 3 µM CuCl_2_. Following wash steps, 325 µl of 0.2% SDS was added to lyse the cells. Cellular copper loading was determined from counts of the cell lysate (450 and 800 keV for 1 min; Packard B5003 gamma counter; Packard BioScience Benelux, Groningen, The Netherlands). For efflux studies, after copper treatment for 22 h as described above, cells were washed four times with Hanks solution containing 3 µM CuCl_2_ before incubation in fresh medium containing no additional copper for 8 h. Cellular copper loading was assessed at 0, 0.5, 1.5, 3, 6 and 8 h as described in the previous paragraph. Copper loading was normalized to the protein concentration of the cell lysate (Bio-Rad Protein Assay Kit; Bio-Rad, The Netherlands). ^65^Zn (PerkinElmer; 1 mCi chloride solution) was used as a control and prepared as described for ^64^Cu experiments. The use of canine and human fibroblasts was approved by the Utrecht University Ethical Committee and the WALTHAM Centre for Pet Nutrition.

#### Statistical analysis

Counts per minute normalized to the protein concentration of the cell lysate (in counts per minute per milligram) were analysed using linear mixed-effect modelling with the R package ‘nlme’. The outcome variable was transformed by taking its natural logarithm in order to fulfil the criteria of normality and constant variance of residuals. Restricted maximum likelihood was used to estimate the fixed effects of duration of ^64^Cu treatment (in hours, modelled as a categorical factor), presence or absence of the ATP7A:p.Thr327Ile substitution, sex (male, female) and their interactions. A stepwise backward method was used to determine the model of best fit, based on Akaike's information criterion. Estimates were reported with their 95% confidence intervals. For the efflux studies, the amount of ^64^Cu at the start of the experiment was set to 100%, and at the time points 0.5, 1.5, 3, 6 and 8 h the amount of ^64^Cu was calculated as a percentage of the initial value. Per time interval, the efflux rate of copper out of the cells was calculated as the percentage efflux per hour. The average efflux rates of fibroblasts derived from dogs with ATP7A wild type and dogs with the ATP7A:p.Thr327Ile substitution were compared using Student's unpaired *t*-test.

#### Western blot

Fibroblasts were treated with 3 µM copper for 0, 6, 22 or 30 h before harvesting. Cell lysates were heated to 70°C for 5 min in loading buffer [50 mM Tris-HCl (pH 6.8), 2% SDS, 0.1% bromophenol blue, 10% glycerol and 40 mM dithiothreitol] and loaded onto preassembled SDS-PAGE. The membrane was blocked with ECL blocking for 1 h, before overnight incubation at 4°C with chicken IgY anti-ATP7A (no. 13995; Abcam) at a dilution of 1:2000 followed by a 1 h incubation at room temperature with the secondary antibody horseradish peroxidase-conjugated goat anti-chicken (no. ab6877; Abcam) at a dilution of 1:40,000 in blocking buffer. In order to show equal protein loading, membranes were western blotted with rabbit anti-tubulin (no. ab6046; Abcam; 1:10,000 dilution) followed by goat anti-rabbit horseradish peroxidase (no. sc2004; Santa Cruz; 1:20,000) and visualized with ECL western blotting detection reagents.

#### Immunofluorescence

The intracellular distribution of GFP-tagged ATP7B or ATP7A proteins was observed 24 or 48 h after transfection. Cells were treated with 500 µM BCS overnight and fixed directly or washed and incubated for 2 h with 200 µM CuSO_4_ before fixation in 4% paraformaldehyde. Then the cells were incubated with blocking/permeabilizing solution (0.5% bovine serum albumin, 0.1% saponin and 50 mM NH__4__Cl in PBS) for 20-30 min. Primary and secondary antibodies were diluted in blocking/permeabilizing solution and added to the cells overnight and for 45 min, respectively. Co-localization between ATP7B variants and organelle markers was visualized using anti-TGN46 antibody (Golgi marker), VAP-A antibody (ER marker), Na^+^/K^+^-ATPase antibody (plasma membrane marker) and LAMP1 antibody (late endosome/lysosome marker) followed by the Alexa Fluor 568 secondary antibody. Antibodies against the tight junction protein occludin were used to label the border between apical and basolateral domains in MDCK cells. To visualize canalicular domains, polarized HepG2 cells expressing ATP7B^WT^ or ATP7B^R1415Q^ were labelled with anti-MRP2 antibody followed by the secondary anti-mouse Alexa Fluor 568. Canine dermal fibroblasts were treated with BCS or CuSO_4_ as described above, fixed and stained for endogenous ATP7A with anti-ATP7A antibodies and with anti-Golgin97 to reveal TGN46. The images were acquired using a 63×1.4 NA oil immersion objective at an LSM710 or LSM700 confocal microscope with appropriate filter sets (Carl Zeiss, Jena, Germany). The intracellular distribution of GFP-tagged ATP7B protein was analysed in 10 randomly selected fields, and the percentage of the cells exhibiting ATP7B in ER, Golgi and/or plasma membrane/vesicles was quantified. A Wilcoxon rank-sum test was used to test differences in percentages of GFP positivity within the ER, Golgi and/or plasma membrane. A *P*-value <0.05 was considered significant. For polarized MDCK cells, confocal *z*-stacks through the entire cell monolayer were taken, and *xz* sections were generated using Zeiss Zen software.
